# Epidemiology and outcomes associated with carbapenem-resistant *Acinetobacter baumannii* and carbapenem-resistant *Pseudomonas aeruginosa:* a retrospective cohort study

**DOI:** 10.1186/s12879-022-07436-w

**Published:** 2022-05-24

**Authors:** Amanda Vivo, Margaret A. Fitzpatrick, Katie J. Suda, Makoto M. Jones, Eli N. Perencevich, Michael A. Rubin, Swetha Ramanathan, Geneva M. Wilson, Martin E. Evans, Charlesnika T. Evans

**Affiliations:** 1 Center of Innovation for Complex Chronic Healthcare (CINCCH), Edward Hines Jr. VA Medical Center, Hines, IL USA; 2 Center of Innovation for Complex Chronic Healthcare (CINCCH), Edward Hines Jr. VA Medical Center, 5000 S. 5th Avenue (151H), Building 1, Room D322, Hines, IL 60141 USA; 3 Department of Medicine, Loyola University Chicago Stritch School of Medicine, Maywood, IL USA; 4 Center for Health Equity Research and Promotion, VA Pittsburgh Health Care System, Pittsburgh, PA USA; 5 School of Medicine, Department of Medicine, University of Pittsburgh, Pittsburgh, PA USA; 6 Department of Veterans’ Affairs, VA Salt Lake City Healthcare System, Salt Lake City, UT USA; 7 Department of Internal Medicine, University of Utah School of Medicine, Salt Lake City, UT USA; 8 Department of Veterans’ Affairs, Iowa City VA Health Care System, Iowa City, IA USA; 9 Department of Internal Medicine, University of Iowa Carver College of Medicine, Iowa City, IA USA; 10 Department of Preventive Medicine, Center for Health Services and Outcomes Research, Northwestern University Feinberg School of Medicine, Chicago, IL USA; 11 MRSA/MDRO Program, VHA National Infectious Diseases Service, VA Central Office and the Lexington VA Medical Center, Lexington, KY USA; 12 Department of Internal Medicine, University of Kentucky School of Medicine, Lexington, KY USA; 13 Preventive Medicine and Center for Health Services and Outcomes Research, Northwestern University, Chicago, IL USA

**Keywords:** Carbapenem resistant organisms, Outcomes, *Acinetobacter baumannii*, *Pseudomonas aeruginosa*

## Abstract

**Background:**

Carbapenem-resistant *Acinetobacter baumannii* (CRAB) and carbapenem-resistant *Pseudomonas aeruginosa* (CRPA) are a growing threat. The objective of this study was to describe CRAB and CRPA epidemiology and identify factors associated with mortality and length of stay (LOS) post-culture.

**Methods:**

This was a national retrospective cohort study of Veterans with CRAB or CRPA positive cultures from 2013 to 2018, conducted at Hines Veterans Affairs Hospital. Carbapenem resistance was defined as non-susceptibility to imipenem, meropenem and/or doripenem. Multivariable cluster adjusted regression models were fit to assess the association of post-culture LOS among inpatient and long-term care (LTC) and to identify factors associated with 90-day and 365-day mortality after positive CRAB and CRPA cultures.

**Results:**

CRAB and CRPA were identified in 1,048 and 8,204 unique patients respectively, with 90-day mortality rates of 30.3% and 24.5% and inpatient post-LOS of 26 and 27 days. Positive blood cultures were associated with an increased odds of 90-day mortality compared to urine cultures in patients with CRAB (OR 6.98, 95% CI 3.55–13.73) and CRPA (OR 2.82, 95% CI 2.04–3.90). In patients with CRAB and CRPA blood cultures, higher Charlson score was associated with increased odds of 90-day mortality. In CRAB and CRPA, among patients from inpatient care settings, blood cultures were associated with a decreased LOS compared to urine cultures.

**Conclusions:**

Positive blood cultures and more comorbidities were associated with higher odds for mortality in patients with CRAB and CRPA. Recognizing these factors would encourage clinicians to treat these patients in a timely manner to improve outcomes of patients infected with these organisms.

**Supplementary Information:**

The online version contains supplementary material available at 10.1186/s12879-022-07436-w.

## Background

In 2015, there were approximately 687,000 hospital acquired infections (HAIs) in United States acute care hospitals and an estimated 72,000 patients died during their hospitalization [1]. Gram-negative organisms such as *Acinetobacter* and *Pseudomonas *spp. have been identified as major causes of nosocomial infections due to their extensive antimicrobial resistance, ability to cause outbreaks, and association with negative outcomes such as increased lengths of stay (LOS) and higher mortality rates [2, 3]. A systematic review identified *Acinetobacter baumannii* and *Pseudomonas aeruginosa* infections had a raw mortality rate of 47% and 23%, respectively [4].

*Acinetobacter baumannii* and *P. aeruginosa* are often multi-drug resistant, including carbapenem resistant [5]. Carbapenem-resistant organisms (CROs), specifically carbapenem-resistant *Pseudomonas aeruginosa* (CRPA), have been identified as critical pathogens due to their enhanced transmissibility and limited treatment options [6]. The 30-day mortality rate of CRPA infections, even with appropriate treatment is 30% [7]. Carbapenem-resistant *A. baumannii* has been listed as a new urgent threat by the Centers for Disease Control and Prevention’s 2019 Antibiotic Resistance Threat Report. In 2017, there were an estimated 8500 cases in hospitalized patients and 700 deaths [8]. In a study of acute care hospitals in the U.S. carbapenem-resistant *Acinetobacter baumannii* (CRAB) and CRPA were found to cause greater than 80% of carbapenem-resistant infections [9]. Those with CRAB infections were found to have longer hospital stays and higher in-hospital mortality compared to matched controls without CRAB infections while those with CRPA had a higher risk for death than those infected with carbapenem-susceptible *P. aeruginosa*. [10, 11] Risk factors for mortality among those with CRAB infections include: a greater number of comorbid conditions at admission and inappropriate antimicrobial treatment [12], as well as site of infection and an intensive care unit (ICU) stay [13]. Severity of illness, ICU stay, and presence of invasive devices were risk factors for mortality for patients with CRPA infections [7, 14].

There is increasing attention on CRAB and CRPA because these organisms can cause HAIs and often have few effective treatment options [15]. While current carbapenem-resistant *Enterobacteriaceae* guidelines are being updated to include these additional CROs, there are little data on CRAB and CRPA epidemiology in VA. The objective of this analysis was to describe the epidemiologic, demographic, medical characteristics, and outcomes of patients with positive cultures with CRAB and CRPA and identify factors associated with mortality and LOS post CRAB and CRPA culture.

## Methods

### Study design, setting, patients

This was a retrospective cohort study of national Veteran Affairs (VA) medical encounter and microbiology laboratory data from adult patients (≥ 18 years) treated at 142 VA facilities from January 1, 2013 to December 31, 2018. Isolates from patients were classified as CRAB and CRPA if a culture from any site grew *A. baumannii* or *A. calcoaceticus *(*A. baumannii* complex*)* or *P. aeruginosa* that was non-susceptible to imipenem, meropenem, and/or doripenem as defined by Clinical Laboratory and Standards Institute (CLSI) minimum inhibitory concentration (MIC) breakpoints. VA guidelines required laboratories to use CLSI M100-S21 or higher, however antibiotic susceptibility testing was performed by each VA laboratory according to their protocol, which may not have been consistent across sites. An electronic survey of 161 Multidrug-resistant Organism (MDRO) Prevention Coordinators at 134 VA Medical Centers (VAMCs) between February 21 and April 30, 2018 found 20.9% of coordinators reported active screening for Carbapenem-resistant *Enterobacterales* (CRE) and carbapenemase-producing-CRE colonization at their site [16]. However, each VA may have variability in surveillance and reporting for CRE [16]. Only cultures identified as CRAB or CRPA were included in the analysis, and only the last culture per patient was included. This analysis was part of a larger quality improvement initiative in VA.

### Data collection

Data were extracted from the VA Corporate Data Warehouse (CDW), a national repository that includes clinical and administrative data from the Veterans Health Administration. The CDW is updated on a continual basis and was used to obtain demographics, clinical setting at the time of culture, microbiology data, comorbidities, previous antibiotic exposure, and facility characteristics where the culture was obtained. Culture specimen type for each culture were categorized into blood, urine, respiratory, and other source (wounds, skin/soft tissue, body fluid, bone/joint, rectal cultures). Comorbidities were identified by ICD-10-CM codes in the 365 days prior to a carbapenem-resistant culture and used to calculate the Charlson comorbidity index. Spinal cord injury and disorder (SCI/D) diagnoses were also identified as the VA serves many Veterans with SCI/D and this population is known to be at risk for MDROs [16]. Previous antibiotic exposures were defined as any antibiotic prescribed in the 90 days before the positive culture. LOS was recorded for the 365 days prior to the positive culture. Facility characteristics included rurality, facility complexity, and geographic region. Facility complexity levels were based on patient characteristics, clinical programs, and teaching programs and categorized as high complexity (1a-c) or low complexity (2–3). Geographic region was based on U.S. Census Bureau categories. Multiple cultures of the same organism from the same patient were excluded, and only the last culture of each patient was included. Outcome variables assessed were 90 day-mortality, 365-day mortality, 30-day mortality for the blood analysis, and post-culture LOS for patients in inpatient and long-term care (LTC).

### Statistical analyses

Descriptive and bivariate statistics were used to summarize patient demographics, medical characteristics, previous healthcare exposure, microbiology data, facility characteristics, and outcomes. The association between CRAB and CRPA and outcomes were assessed using Students t-test, ANOVA, and Chi-square tests. Unadjusted and adjusted negative binomial models were used to assess post-culture LOS among inpatients and those in LTC. Post-culture LOS was modeled as a count variable using a negative-binomial model due to overdispersion and unadjusted and adjusted incidence rate ratios (IRRs) and 95% CIs are presented. Generalized estimating equations logistic regression was used to identify factors associated with 90-day and 365-day mortality after positive CRAB and CRPA cultures and a sensitivity analysis was performed with patients with SCI/D. A separate analysis was conducted to identify factors associated with 90-day and 365-day mortality of CRAB and CRPA blood cultures and a sensitivity model was run with 30-day mortality. The most parsimonious models were presented with adjusted odds ratios (ORs) or IRRs and 95% CIs, which included covariates significant at the 0.05 level. Analyses and modeling were carried out using SAS, version 9.4 (SAS Institute).

## Results

**Fig. 1:**
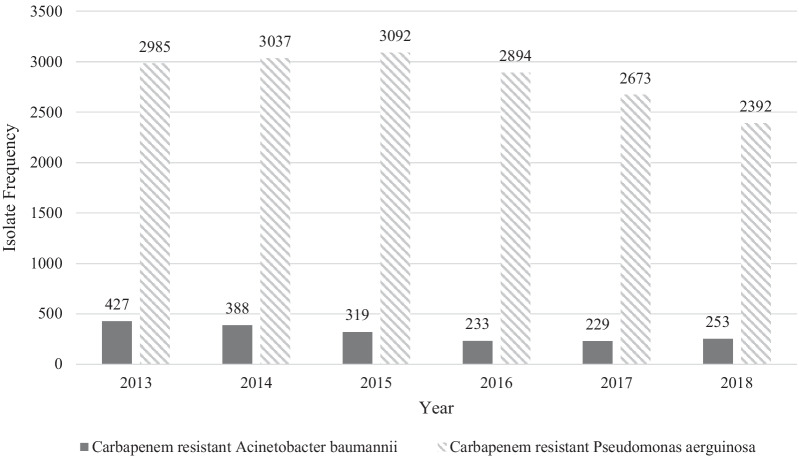
Carbapenem-resistant *Acinetobacter baumannii* and carbapenem-resistant *Pseudomonas aeruginosa Total* isolates by year

Between January 1, 2013 and December 31, 2018, 1849 cultures from 1048 unique patients grew CRAB and 17,073 cultures from 8204 unique patients grew CRPA. From 2013 to 2018, the number of CRAB and CRPA cultures decreased by 20% and 41% respectively. Figure 1 shows the number of total isolates in the total population which indicates trends in CRPA cultures initially increased through 2015, followed by decreases through 2018, while CRAB cultures decreased from 2013 to 2017, then increased in 2018 (Fig. 1).

Demographics, facility characteristics, and medical characteristics of patients with CRAB, are shown in Table 1. Patients with CRAB were mostly white, non-Latine males and had an average age of 68 (sd = 11.6) years. The majority of patients were from the southern region (49.1%) of the United States and were from urban (95.9%), high complexity (97.8%) facilities. More than half of patients with CRAB were from inpatient care settings, followed by outpatient (25.1%) and then LTC (21.7%). CRAB cultures were primarily from other specimens (41.4%), followed by urine specimens (29.3%). The average Charlson score was 5.0. Approximately a third had any antibiotic exposure in the 90 days prior to the culture date (34.0%).

**Table 1:** Bivariate and multivariable analysis of CRAB cultures evaluating association between patient/facility characteristics and 90-day mortality

Characteristics [N = 1048]	Death 90 days after culture date (%) [N = 318]	No death 90 days after culture date (%) [N = 730]	Unadjusted odds ratio (95% CI)	Adjusted odds ratio (95% CI)
Demographic and clinical characteristics				
Male	315 (99.1)	709 (97.1)	3.11 (0.92–10.5)	–
Age category				
18–49	3 (0.9)	50 (6.9)	Reference	Reference
50–64	61 (19.2)	258 (35.3)	**3.92 (1.19–13.05)**	3.24 (0.93–11.34)
65 +	254 (79.9)	422 (57.8)	**10.03 (3.10–32.5)**	**7.41 (2.16–25.37)**
Race				
White	200 (62.9)	455 (62.3)	Reference	–
African American	112 (35.1)	263 (36.0)	0.97 (0.74–1.28)	–
Other^*^	6 (1.9)	12 (1.6)	1.14 (0.42–3.07)	–
Ethnicity				
Non-Latine	260 (81.8)	673 (92.2)	Reference	–
Latine	58 (18.2)	57 (7.8)	**2.63 (1.78–3.90)**	–
Region				
South	139 (43.7)	375 (51.4)	Reference	Reference
Midwest	40 (12.6)	133 (18.2)	0.81 (0.54–1.22)	0.77 (0.49–1.21)
West	65 (20.4)	111 (15.2)	**1.58 (1.10–2.27)**	1.21 (0.81–1.82)
Northeast	22 (6.9)	71 (9.7)	0.84 (0.50–2.40)	0.59 (0.34–1.05)
U.S. Territory	52 (16.4)	40 (5.5)	**3.51 (2.22–5.53)**	**2.03 (1.19–3.45)**
Rurality				
Urban	308 (96.9)	697 (95.5)	Reference	–
Rural	10 (3.1)	33 (4.5)	0.69 (0.33–1.41)	–
Facility complexity				
High	315 (99.1)	710 (97.3)	Reference	–
Low	3 (0.9)	20 (2.7)	0.34 (0.10–1.15)	–
Care setting				
Outpatient	61 (19.2)	202 (27.7)	Reference	Reference
Long-term care	26 (8.2)	201 (27.5)	**0.43 (0.26–0.71)**	**0.30 (0.16–0.54)**
Inpatient	231 (72.6)	327 (44.8)	**2.34 (1.68–3.26)**	**1.59 (1.09–2.32)**
Specimen type				
Urine	50 (15.7)	257 (25.2)	Reference	Reference
Respiratory^‡^	124 (39.0)	127 (17.4)	**5.02 (3.39–7.42)**	**3.84 (2.48–5.93)**
Blood	36 (11.3)	20 (2.7)	**9.25 (4.95–17.28)**	**6.98 (3.55–13.73)**
Other^†^	108 (34.0)	326 (44.7)	**1.70 (1.17–2.47)**	**1.68 (1.12–2.52)**
Co-morbidities				
Mean Charlson Score (SD)	6 (3.2)	5 (2.8)	** < 0.0001**^**§**^	**1.11 (1.05–1.17)**
SCI/D				
No	272 (85.5)	482 (66.0)	Reference	–
Yes	46 (14.5)	248 (34.0)	**0.33 (0.23–0.47)**	–
Antibiotic exposure in previous 90 days				
Any antibiotics				–
No	230 (72.3)	462 (63.3)	Reference	Reference
Yes	88 (27.7)	268 (36.7)	**0.66 (0.50–0.88)**	**0.65 (0.46–0.91)**
Fluoroquinolones				
No	274 (86.2)	593 (81.2)	Reference	–
Yes	44 (13.8)	137 (18.8)	0.70 (0.48–1.01)	–
3rd and 4th generation cephalosporins				
No	311 (97.8)	712 (97.5)	Reference	–
Yes	7 (2.2)	18 (2.5)	0.89 (0.37–2.15)	–
Carbapenems				
No	316 (99.4)	725 (99.3)	Reference	–
Yes	2 (0.6)	5 (0.7)	0.92 (0.18–4.76)	–
Length of stay 1 year prior to culture				
Mean, median (SD)	75, 25 (95.5)	67, 31 (89.7)	0.1831^§^	1.00 (1.00–1.00)

Significant associations are shown in bold (p < 0.05)

^*^Other includes, Asian, Native American and Pacific Islander

^†^Other specimen types include wounds, skin/soft tissue, body fluid, bone/joint, rectal cultures

^‡^Respiratory specimen types include bronchial alveolar, tracheal aspirate, induced sputum, etc.

^§^T-test p-value

Among patients with CRAB, 30.3% died within 90 days and 46.5% died within 365 days after a positive culture. Table 1 shows unadjusted associations between demographic and medical characteristics and death within 90 days for CRAB. In the final adjusted models, being in the inpatient setting, older age, having a blood, respiratory, or other specimen, being in a U.S. territory, and higher Charlson score were associated with a higher odds of 90-day mortality (Table 1). Patients with previous antibiotic exposure in the past 90 days and in LTC were associated with lower odds of 90-day mortality compared to cultures from outpatient care. Additional file 1: Table S1 shows similar findings for factors associated with 365-day mortality, however patients from the Midwest region were associated with decreased odds of 365-day mortality. In the sensitivity analysis of only patients with SCI/D, similar results were found for 90-day mortality. However, age, region, LTC, other specimen, Charlson score, and previous antibiotic exposure were no longer significant.

Among patients with CRAB cultures taken in the inpatient setting, the median LOS post-culture was 20 days (range 0–365). Factors significantly associated with increased post-LOS in adjusted inpatient models included location in the Midwest, West, or U.S. Territory. Factors significantly associated with decreased post-LOS in adjusted inpatient models included higher Charlson score, blood culture, and older age. For CRAB cultures taken in the LTC setting the median LOS post culture was 96 days (range 0–365). In the adjusted LTC models any antibiotic exposure in the previous 90 days was associated with a decreased LOS, while other specimen cultures (wounds, skin/soft tissue, body fluid, bone/joint, rectal cultures) were associated with an increased LOS (Table 2).

**Table 2:** Risk factors associated with LOS among inpatient and LTC post-CRAB culture: Unadjusted and multivariable regression

Characteristics	Inpatient [N = 558]		Long-Term Care (LTC) [N = 227]	
	Unadjusted IRR (95% CI)	Adjusted IRR (95% CI)	Unadjusted IRR (95% CI)	Adjusted IRR (95% CI)
Demographic & clinical characteristics				
Male	0.58 (0.28–1.21)	–	0.73 (0.32–1.65)	–
Age category				
18–49	Reference	Reference	Reference	Reference
50–64	0.68 (0.39–1.19)	0.64 (0.37–1.10)	1.11 (0.68–1.80)	1.04 (0.63–1.71)
65 +	**0.49 (0.29–0.84)**	**0.50 (0.29–0.84)**	0.98 (0.61–1.58)	0.91 (0.56–1.49)
Race				
White	Reference	–	Reference	–
African American	1.07 (0.86–1.33)	–	1.02 (0.78–1.34)	–
Other*	0.68 (0.30–1.54)	–	0.67 (0.27–1.65)	–
Ethnicity				
Non-Latine	Reference	–	Reference	–
Latine	1.01 (0.75–1.37)	–	1.05 (0.57–1.94)	–
Region				
South	Reference	Reference	Reference	–
Midwest	1.27 (0.94–1.71)	**1.41 (1.05–1.88)**	0.81 (0.56–1.16)	–
West	1.14 (0.86–1.52)	**1.35 (1.02–1.79)**	1.23 (0.83–1.83)	–
Northeast	0.79 (0.55–1.13)	0.98 (0.69–1.39)	1.13 (0.64–2.01)	–
U.S. Territory	1.16 (0.82–1.64)	**1.49 (1.04–2.13)**	1.18 (0.52–2.68)	–
Rurality				
Urban	Reference	–	Reference	–
Rural	0.74 (0.47–1.15)	–	1.01 (0.32–3.19)	–
Facility complexity				
High	Reference	–	Reference	–
Low	0.51 (0.22–1.16)	–	1.53 (0.63–3.74)	–
Specimen type				
Urine	Reference	Reference	Reference	Reference
Respiratory^‡^	0.95 (0.71–1.26)	0.82 (0.62–1.10)	1.31 (0.90–1.91)	1.23 (0.84–1.79)
Blood	**0.42 (0.27–0.67)**	**0.47 (0.30–0.73)**	1.11 (0.48–2.58)	1.15 (0.51–2.61)
Other^†^	1.04 (0.79–1.37)	1.04 (0.80–1.36)	1.24 (0.92–1.68)	**1.34 (1.00–1.81)**
Co-morbidities				
Charlson Score	**0.96 (0.93–0.99)**	**0.95 (0.92–0.98)**	1.01 (0.96–1.05)	0.99 (0.94–1.04)
SCI/D				
No	Reference	–	Reference	–
Yes	**1.55 (1.14–2.09)**	–	0.88 (0.65–1.19)	–
Antibiotic exposure in previous 90 days				
Any Antibiotics				
No	Reference	–	Reference	Reference
Yes	0.98 (0.79–1.22)	–	**0.67 (0.49–0.93)**	**0.72 (0.52–0.99)**
Fluoroquinolones				
No	Reference	–	Reference	–
Yes	0.93 (0.70–1.23)	–	**0.46 (0.30–0.69)**	–
3rd and 4th generation cephalosporins				
No	Reference	–	Reference	–
Yes	1.48 (0.77–2.85)	–	0.62 (0.20–1.97)	–
Carbapenems				
No	Reference	–	Reference	–
Yes	1.37 (0.41–4.65)	–	–	–
Length of stay 1 year prior to culture	**1.00 (1.00–1.00)**	**1.00 (1.00–1.01)**	**1.00 (1.00–1.00)**	**1.00 (1.00–1.00)**

Significant associations are shown in bold (p < 0.05)

^*^Other includes, Asian, Native American and Pacific Islander

^†^Other specimen types include wounds, skin/soft tissue, body fluid, bone/joint, rectal cultures

^‡^Respiratory specimen types include bronchial alveolar, tracheal aspirate, induced sputum, etc.

Patients with CRPA were mostly white, non-Latine males with an average age of 70 (sd = 12.4) years. Most isolates were from the southern region (41.4%) and were from urban (92.9%), high complexity (91.2%) facilities (Table 3). Most cultures were from the inpatient setting (44.4%), followed by the outpatient setting (42.8%) and LTC (12.7%). Over half of the cultures were from urine specimens (52.7%). The average Charlson score for patients with CRPA was 4.6 and approximately 40.8% had an antibiotic exposure in the past 90 days.

**Table 3:** Bivariate and multivariable analysis of CRPA cultures evaluating association between patient/facility characteristics and 90-day mortality

Characteristics [n = 8204]	Death 90 days after culture date (%) [N = 2012]	No death 90 days after culture date (%) [N = 6192]	Unadjusted odds ratio (95% CI)	Adjusted odds ratio (95% CI)
Demographic and clinical characteristics				
Male	1983 (98.6)	6019 (97.2)	**1.97 (1.32–2.92)**	–
Age category				
18–49	36 (1.8)	354 (5.7)	Reference	Reference
50–64	378 (18.8)	1516 (24.5)	**2.45 (1.71–3.52)**	**1.83 (1.24–2.69)**
65 +	1598 (79.4)	4322 (69.8)	**3.63 (2.57–5.14)**	**2.96 (2.04–4.29)**
Race				
White	1514 (75.3)	4908 (79.3)	Reference	–
African American	469 (23.3)	1181 (19.1)	**1.29 (1.15–1.45)**	–
Other*	29 (1.4)	103 (1.7)	0.91 (0.60–1.38)	–
Ethnicity				
Non-Latine	1799 (89.4)	5737 (92.7)	Reference	–
Latine	213 (10.6)	455 (7.3)	**1.49 (1.26–1.77)**	–
Region				
South	861 (42.8)	2533 (40.9)	Reference	Reference
Midwest	370 (18.4)	1334 (21.5)	**0.82 (0.71–0.94)**	**0.77 (0.66–0.90)**
West	391 (19.4)	1377 (22.2)	**0.84 (0.73–0.96)**	**0.85 (0.73–0.99)**
Northeast	242 (12.0)	707 (11.4)	1.01 (0.95–1.19)	**0.83 (0.69–1.00)**
U.S. Territory	148 (7.4)	241 (3.9)	**1.81 (1.45–2.25)**	1.23 (0.96–1.58)
Rurality				
Urban	1892 (94.0)	5733 (92.6)	Reference	–
Rural	120 (6.0)	459 (7.4)	**0.79 (0.64–0.98)**	–
Facility complexity				
High	1908 (94.8)	5578 (90.1)	Reference	–
Low	104 (5.2)	614 (9.9)	**0.50 (0.40–0.61)**	–
Care setting				
Outpatient	398 (19.8)	3117 (50.3)	Reference	Reference
Long-term care	205 (10.2)	838 (13.5)	**1.92 (01.59–2.31)**	1.08 (0.86–1.36)
Inpatient	1409 (70.0)	2237 (36.1)	**4.93 (4.36–5.58)**	**2.95 (2.58–3.38)**
Specimen type				
Urine	758 (37.7)	3565 (57.6)	Reference	Reference
Respiratory^‡^	823 (40.9)	936 (15.1)	**4.12 (3.66–4.56)**	**2.58 (2.25–2.96)**
Blood	91 (4.5)	93 (1.5)	**4.60 (3.41–6.21)**	**2.82 (2.04–3.90)**
Other^†^	340 (16.9)	1598 (25.8)	1.00 (0.87–1.15)	**0.84 (0.72–0.98)**
Co-morbidities				
Mean Charlson Score (SD)	6 (3.1)	4 (2.9)	** < 0.0001**^**§**^	**1.13 (1.11–1.15)**
SCI/D				
No	1813 (90.1)	5083 (82.1)	Reference	Reference
Yes	199 (9.9)	1109 (17.9)	**0.50 (0.43–0.59)**	**0.64 (0.53–0.77)**
Antibiotic exposure in previous 90 days				
Any antibiotics				
No	1409 (70.0)	3445 (55.6)	Reference	Reference
Yes	603 (30.0)	2747 (44.40	**0.54 (0.48–0.60)**	**0.58 (0.52–0.66)**
Fluoroquinolones				
No	1765 (87.7)	5098 (82.3)	Reference	–
Yes	247 (12.3)	1094 (17.7)	**0.65 (0.56–0.76)**	–
3rd and 4th generation cephalosporins				
No	1960 (97.4)	5971 (96.4)	Reference	–
Yes	52 (2.6)	221 (3.6)	**0.72 (0.53–0.97)**	–
Carbapenems				
No	1999 (99.4)	6163 (99.5)	Reference	–
Yes	13 (0.6)	29 (0.5)	1.38 (0.72–2.66)	–
Length of stay 1 year prior to culture				
Mean, median (SD)	73, 38 (92.1)	42, 10 (75.6)	** < 0.0001**^**§**^	**1.00 (1.00–1.00)**

Significant associations are shown in bold (p < 0.05)

^*^Other includes, Asian, Native American and Pacific Islander

^†^Other specimen types include wounds, skin/soft tissue, body fluid, bone/joint, rectal cultures

^‡^Respiratory specimen types include bronchial alveolar, tracheal aspirate, induced sputum, etc.

^§^t-test p-value

About 24.5% of patients died within 90 days and 40.3% of patients died within 365 days after a positive CRPA culture. Table 3 shows unadjusted associations between demographic and medical characteristics and death within 90 days for CRPA (Table 3). In multivariable adjusted analyses older age, higher Charlson score, respiratory and blood cultures, and inpatient settings were associated with higher odds of death at 90 days after CRPA culture (Table 3). Any antibiotic exposure in the previous 90 days, other specimens, being in the Midwest, West, or Northeast region, and being a patient with SCI/D had lower odds of 90-day mortality. Similar findings were seen for 365-day mortality, however LTC was associated with increased odds for 365-day mortality and location in the Midwest was associated with decreased odds for 365-day mortality (Additional file 1: Table S2). In the sensitivity analysis with patients with SCI/D, similar results were found for 90-day mortality. However, other specimen types, West, and Northeast region were no longer significant, while LTC was associated with a decreased odds for 90-day mortality.

Among patients with CRPA in an inpatient setting, the median LOS post-culture was 16 days (range 0–365). Factors significantly associated with increased post-culture LOS in adjusted inpatient models included other specimens, SCI/D and higher Charlson score, and being in the West U.S. region. Older age, blood, and respiratory specimen types were associated with a decreased post-LOS (Table 4). For CRPA cultures taken in the LTC setting, the median LOS post culture was 70 days (range 0–365). In the unadjusted & adjusted LTC models other specimen type was associated with an increased post LOS.

**Table 4:** Risk factors associated with LOS among inpatient and LTC post-CRPA culture: unadjusted and multivariable regression

Characteristics	Inpatient [N = 3646]			Long-term care (LTC) [N = 1043]
	Unadjusted IRR (95% CI)	Adjusted IRR (95% CI)	Unadjusted IRR (95% CI)	Adjusted IRR (95% CI)
Demographic and clinical characteristics				
Male	0.78 (0.59–1.03)	–	1.39 (0.90–2.13)	–
Age category				
18–49	Reference	Reference		Reference
50–64	0.81 (0.65–1.01)	**0.73 (0.59–0.90)**	1.17 (0.88–1.55)	1.06 (0.81–1.40)
65 +	**0.60 (0.49–0.74)**	**0.57 (0.46–0.70)**	1.07 (0.82–1.40)	0.99 (0.76–1.28)
Race				
White	Reference	–	Reference	–
African American	**1.14 (1.03–1.25)**	–	0.99 (0.84–1.16)	–
Other*	1.11 (0.81–1.52)	–	0.97 (0.56–1.67)	–
Ethnicity				
Non-Latine	Reference	–	Reference	–
Latine	0.91 (0.80–1.04)	–	0.93 (0.71–1.22)	–
Region				
South	Reference	Reference	Reference	–
Midwest	1.04 (0.93–1.15)	0.99 (0.89–1.09)	0.88 (0.74–1.05)	–
West	**1.24 (1.11–1.38)**	**1.25 (1.13–1.39)**	1.01 (0.83–1.23)	–
Northeast	1.01 (0.89–1.15)	1.07 (0.95–1.21)	0.82 (0.64–1.05)	–
U.S. Territory	0.99 (0.83–1.17)	1.14 (0.97–1.34)	0.84 (0.60–1.19)	–
Rurality				
Urban	Reference	–	Reference	–
Rural	0.90 (0.77–1.06)	–	1.12 (0.83–1.51)	–
Facility complexity				
High	Reference	–	Reference	–
Low	**1.21 (1.02–1.45)**	–	1.04 (0.80–1.33)	–
Specimen type				
Urine	Reference	Reference	Reference	Reference
Respiratory^‡^	**0.81 (0.74–0.89)**	**0.76 (0.69–0.83)**	0.98 (0.81–1.19)	0.97 (0.80–1.17)
Blood	**0.63 (0.50–0.78)**	**0.67 (0.54–0.83)**	1.24 (0.73–2.12)	1.02 (0.60–1.72)
Other^†^	**1.29 (1.16–1.43)**	**1.27 (1.15–1.40)**	**1.36 (1.15–1.61)**	**1.38 (1.17–1.63)**
Co-morbidities				
Charlson Score	**1.02 (1.00–1.03)**	**1.01 (1.00–1.02)**	1.01 (0.99–1.04)	0.99 (0.97–1.02)
SCI/D				
No	Reference	Reference	Reference	–
Yes	**1.87 (1.62–2.16)**	**1.43 (1.23–1.65)**	1.01 (0.88–1.16)	–
Antibiotic exposure in previous 90 days				
Any antibiotics				
No	Reference	–	Reference	Reference
Yes	**0.85 (0.79–0.93)**	–	**0.80 (0.68–0.95)**	1.00 (0.84–1.19)
Fluoroquinolones				
No	Reference	–	Reference	–
Yes	**0.82 (0.73–0.92)**	–	**0.64 (0.49–0.83)**	–
3rd and 4th generation cephalosporins				
No	Reference	–	Reference	–
Yes	1.11 (0.88–1.41)	–	0.80 (0.52–1.23)	–
Carbapenems				
No	Reference	–	Reference	–
Yes	1.05 (0.66–1.67)	–	1.51 (0.31–7.37)	–
Length of stay 1 year prior to culture				
Mean, median (SD)	**1.00 (1.00–1.00)**	**1.00 (1.00–1.00)**	**1.00 (1.00–1.00)**	**1.00 (1.00–1.00)**

Significant associations are shown in bold (p < 0.05)

^*^Other includes, Asian, Native American and Pacific Islander

^†^Other specimen types include wounds, skin/soft tissue, body fluid, bone/joint, rectal cultures

^‡^Respiratory specimen types include bronchial alveolar, tracheal aspirate, induced sputum, etc.

In the combined analysis, 32.3% of patients with CRAB or CRPA blood cultures died within 30 days after the positive culture, 45.5% died within 90 days, and 60.7% died within 365 days. Among patients with a CRAB blood culture, 58.9% died within 90 days, while 42.6% of patients with a CRPA blood culture died within 90 days post-culture (Additional file 1: Table S3). In multivariable adjusted analyses, higher Charlson score was associated with an increased odds of 90-day mortality, while being in LTC, having exposure to antibiotics in the past 90 days, and having a CRPA culture were associated with a decreased odds of 90-day mortality. Similar findings were seen for 365-day mortality, but inpatient care had increased odds of mortality (Additional file 1: Table S4). A sensitivity analysis with 30-day mortality found similar results to the 90-day model, however antibiotic exposure in the past 90 days was insignificant and being in an inpatient setting was significantly associated with increased odds of 30-day mortality. When looking at CRPA and CRAB blood cultures from patients in the inpatient setting, the median LOS post-culture was 19 (range 0–365). In LTC, the median LOS post-culture was 110 (range 0–365).

## Discussion

In this national analysis of Veterans, CRPA infections occurred more often than CRAB infections, but the overall frequency of both CROs decreased from 2013 to 2018. The CRAB decrease occurred after publication of the CDC’s Antimicrobial Resistance Report. Similar trends were observed in a national incidence study from 2012 to 2017, where CRAB and multi drug-resistant *Pseudomonas aeruginosa* (MDRPA) decreased [8, 18]. These decreases may be a result of implementation of infection prevention and antimicrobial stewardship interventions during the study time frame. However, we did not evaluate the percentage of *Acinetobacter* or *Pseudomonas spp.* that were carbapenem-susceptible, thus we were unable to determine whether the trend was seen in carbapenem susceptible cultures. Despite this downward trend in frequency, CRAB and CRPA infections should continue to be considered a serious threat. These CROs are frequently resistant to many other antimicrobial treatments in addition to carbapenems which makes CRAB and CRPA difficult to treat with available antibiotics [8]. Among patients with CRAB, 30.3% died within 90 days, increasing to 46.5% after 365 days. The proportion of patients with CRAB that died within 90 days was less than other studies measuring 90-day and 30-day mortality [12, 19]. This may be a result of implementation of infection prevention and antimicrobial stewardship during the study period that may have contributed to declines in incidence and mortality [20]. Among patients with CRPA, 24.5% died within 90 days, increasing to 40.3% 365 days post culture. The proportion of CRPA patients that died were less than those reported for MDRPA and carbapenem resistant gram-negative bacteria infections in the bloodstream [21]. In patients with positive blood cultures of CRAB and CRPA, 45.5% of patients died within 90 days, which is slightly higher than the reported 38% mortality rate for carbapenem resistant gram-negative bacteria infections in the bloodstream [22].

Among patients with CRAB, a higher Charlson score was associated with increased odds of 90-day and 365-day mortality. This agrees with other studies that found underlying medical conditions were risk factors for mortality [12, 23]. Additionally, a non-urine culture was also associated with increased odds of death, with blood cultures having higher odds than respiratory or other specimens. This may be due to the likelihood that cultures from urine reflect colonization rather than true infection. Bacteremia typically results in greater severity of illness which may lead to septic shock, which has been found to be a risk factor for mortality [23]. A separate study had shown that 95% of deaths attributable to sepsis occurred within 28 days after a positive blood culture [24]. In the sensitivity analysis restricted only to patients with SCI/D, factors associated with 90-mortality were found to be similar to the overall CRAB cohort, suggesting that there are no differences in risk factors for mortality for patients with SCI/D and CRAB and patients with CRAB.

In patients with CRPA, results agreed with previous findings that inpatient care setting is a risk factor for mortality [14]. Patients with CRPA had increased odds for mortality with blood and respiratory cultures and with increased comorbidities, which is consistent with other reports [25]. An interesting finding was that antimicrobial exposure in the previous 90 days was associated with decreased odds for death. This is unusual because prior studies have shown that previous antibiotic exposure was associated with acquisition of MDROs, and MDROs have been associated with higher odds of mortality [26, 27]. This may be because these patients are in areas with high MDRO exposure and acquired CRPA without traditional risk factors. It is also possible that patients had a previous infection with a different organism and received an antibiotic which successfully eliminated the infection. Additionally, patients with SCI/D were associated with decreased odds for death. This may be due to factors unique to the immunology of patients with SCI/D, where they can have increased immunity against bacterial invasion due to repeated infections [28]. In the sensitivity analysis restricted only to patients with SCI/D, antibiotic exposure in the prior 90 days and LTC compared to outpatient care were associated with decreased odds of mortality. This may be a result of patients with SCI/D being in LTC and receiving more care overall and having less acute illnesses.

This analysis found associations with decreased post-culture LOS for older age and blood specimen types from inpatient settings. This may be a result of those in inpatient care having more severe illness, such as septicemia, and being at a higher risk for mortality, which would reduce their LOS. However, in LTC settings other specimen types were associated with an increased LOS. This may be a result of skin/soft tissue and wound cultures representing colonization, as LTC facilities can act as reservoirs for MDROs, which can lead to infection and increased LOS [29]. Interestingly, any previous antibiotic exposure in the past 90 days was associated with decreased LOS in LTC cultures among patients with CRAB but was insignificant among patients with CRPA. This may be a result of the antibiotic exposure variable lacking granularity. It is possible that some of the antibiotics received in the past 90 days may have been empirical treatment for the cultures taken and had activity against the bacteria.

In the analysis of CRPA and CRAB blood cultures, results agreed with previous studies that found older age and higher Charlson score associated with increased odds for mortality [22]. In the adjusted analysis blood infection with *Pseudomonas* was associated with a decreased odds for mortality compared to blood infection with *Acinetobacter*. This agrees with other studies that found *Acinetobacter* to have higher rates and increased odds of mortality than *Pseudomonas* [4, 9]*.* Interestingly, being in LTC had decreased odds for 90-day mortality, while inpatient care had increased odds for 30-day mortality. This may be because those with bacteria in their blood are sicker and are more likely to be in inpatient care.

This analysis had several limitations. First, a comparator group of carbapenem-susceptible *Acinetobacter baumannii* or *Pseudomonas aeruginosa* cultures were not included and thus did not identify risk factors associated with carbapenem resistance. Second, this analysis did not incorporate treatment, so we were unable to examine how treatment affected outcomes. Third, we did not have access to which specific CLSI breakpoints VA microbiology labs used which may have altered the identification of bacterial susceptibilities. Additionally, we did not capture other resistance patterns and were unable to measure the how difficult to treat these isolates may have been. We also were unable to identify if patients died as inpatient, LTC patients, or as outpatients and therefore unable to assess if early inpatient deaths reduced LOS post-culture. Finally, we were unable to determine colonization vs infection. However, this is the largest analysis evaluating clinical outcomes of patients in VA with CRAB and CRPA cultures.

## Conclusions

CRPA isolates occurred more often than CRAB isolates in the VA, but both decreased from 2013 to 2018. Patients with CRAB had a higher proportion of deaths within 90 days. Additionally, having a blood or respiratory culture and more comorbidities were associated with higher odds for 90-day and 365-day mortality in patients with CRPA and CRAB. Therefore, it is important for clinicians to recognize these factors in patients infected with these CROs and to appropriately treat these patients in a timely manner to cease the transmission and improve outcomes of patients infected with these pathogens.

## Supplementary Information

**Additional file 1: Table S1.** Bivariate and multivariable analysis of CRAB cultures evaluating association between patient/facility characteristics and 365-day mortality. **Table S2.** Bivariate and multivariable analysis of CRPA cultures evaluating association between patient/facility characteristics and 365-day mortality. **Table S3.** Bivariate/multivariable analysis of CRAB/CRPA blood cultures evaluating association between patient/facility characteristics and 90-day/30-day mortality. **Table S4.** Bivariate/multivariable analysis of CRAB/CRPA blood cultures evaluating association between patient/facility characteristics and 365-day mortality.

## Data Availability

We are committed to collaborating and sharing these data to maximize their value to improve Veterans and others’ health and health care, to the greatest degree consistent with current Veterans Administration regulations and policy. We can provide access to the programming code used to identify the sample and conduct analyses. We cannot provide a link to the database directly as it would compromise patients’ anonymity, and permissions from VA are needed to obtain the data.

## Abbreviations

HAIs: Hospital acquired infections
LOS: Length of stay
CROs: Carbapenem-resistant organisms
CRPA: Carbapenem-resistant *Pseudomonas aeruginosa*
CRAB: Carbapenem-resistant *Acinetobacter baumannii*
ICU: Intensive care unit
VA: Veterans Affairs
CLSI: Clinical Laboratory and Standards Institute
MIC: Minimum inhibitory concentration
CRE: Carbapenem-resistant *Enterobacterales*
MDRO: Multidrug-resistant organism
VAMCs: Veteran Affairs Medical Centers
CDW: Corporate Data Warehouse
SCI/D: Spinal cord injury and disorder
LTC: Long-term care
IIR: Incidence rate ratios
OR: Odds ratio
MDRPA: Multidrug-resistant *Pseudomonas aeruginosa*
