# Supplementary material for: Epidemiology and outcomes associated with carbapenem-resistant Acinetobacter baumannii and carbapenem-resistant Pseudomonas aeruginosa: a retrospective cohort study
Source: BMC Infect Dis. 2022 May 24;22:491. doi: 10.1186/s12879-022-07436-w (PMC9128216; doi:10.1186/s12879-022-07436-w)
Supplement: Supplementary file 1 — Additional file 1: Table S1. Bivariate and multivariable analysis of CRAB cultures evaluating association between patient/facility characteristics and 365-day mortality. Table S2. Bivariate and multivariable analysis of CRPA cultures evaluating association between patient/facility characteristics and 365-day mortality. Table S3. Bivariate/multivariable analysis of CRAB/CRPA blood cultures evaluating association between patient/facility characteristics and 90-day/30-day mortality. Table S4. Bivariate/multivariable analysis of CRAB/CRPA blood cultures evaluating association between patient/facility characteristics and 365-day mortality. [file 12879_2022_7436_MOESM1_ESM.docx]

| Supplemental Table 1. Bivariate and multivariable analysis of CRAB cultures evaluating association between patient/facility characteristics and 365-day mortality | | | | |
| --- | --- | --- | --- | --- |
| Characteristics  [N=1048] | Death 365  Days  After Culture  Date  (%) [N=487] | No Death 365  Days After  Culture Date (%) [N=561] | Unadjusted  Odds  Ratio  (95% CI) | Adjusted Odds  Ratio  (95% CI) |
| *Demographic & Clinical Characteristics* |  |  |  |  |
| Male | 481 (98.8) | 543 (97.8) | **2.66 (1.05-6.75)** | --- |
| Age category |  |  |  |  |
| 18-49 | 7 (1.4) | 46 (8.2) | Reference | Reference |
| 50-64 | 118 (24.2) | 201 (35.8) | **3.85 (1.69-8.81)** | **3.55 (1.44-8.71)** |
| 65+ | 362 (74.3) | 314 (56.0) | **7.57 (3.37-17.0)** | **6.27 (2.59-**  **15.16)** |
| Race |  |  |  |  |
| White | 307 (63.0) | 348 (62.0) | Reference | --- |
| African American | 171 (35.1) | 204 (36.4) | 0.95 (0.74-1.23) | --- |
| Other* | 9 (1.9) | 9 (1.6) | 1.13 (0.45-2.89) | --- |
| Ethnicity |  |  |  |  |
| Non-Latine | 415 (85.2) | 518 (92.3) | Reference |  |
| Latine | 72 (14.8) | 43 (7.7) | **2.09 (1.40-3.12)** | --- |
| Region |  |  |  |  |
| South | 225 (46.2) | 289 (51.5) | Reference | Reference |
| Midwest | 60 (12.3) | 113 (20.1) | **0.68 (0.47-0.98)** | **0.64 (0.43-0.96)** |
| West | 93 (19.1) | 83 (14.8) | **1.43 (1.02-2.03)** | 1.11 (0.76-1.64) |
| Northeast | 45 (9.2) | 48 (8.6) | 1.20 (0.77-1.87) | 0.96 (0.58-1.59) |
| U.S. Territory | 64 (13.1) | 28 (5.0) | **2.94 (1.82-4.73)** | 1.74 (1.00-3.02) |
| Rurality |  |  |  |  |
| Urban | 467 (95.9) | 538 (95.9) | Reference | --- |
| Rural | 20 (4.1) | 23 (4.1) | 1.00 (0.54-1.85) | --- |
| Facility Complexity |  |  |  |  |
| High | 477 (98.0) | 548 (97.7) | Reference | --- |
| Low | 10 (2.0) | 13 (2.3) | 0.88 (0.38-2.04) | --- |
| Care Setting |  |  |  |  |
| Outpatient | 96 (19.7) | 167 (29.8) | Reference | Reference |
| Long-term care | 65 (13.4) | 162 (28.9) | 0.70 (0.48-1.02) | **0.53 (0.32-0.85)** |
| Inpatient | 326 (66.9) | 232 (41.3) | **2.44 (1.81-3.31)** | **1.63 (1.16-2.30)** |
| Specimen Type |  |  |  |  |
| Urine | 91 (18.7) | 216 (38.5) | Reference | Reference |
| Respiratory^‡^ | 182 (37.4) | 69 (12.3) | **6.26 (4.33-9.06)** | **4.95 (3.29-7.45)** |
| Blood | 45 (9.2) | 11 (2.0) | **9.71 (4.81-**  **19.62)** | **7.58 (3.60-**  **15.97)** |
| Other^†^ | 169 (34.7) | 265 (47.2) | **1.51 (1.11-2.07)** | **1.46 (1.04-2.05)** |
| Co-Morbidities |  |  |  |  |
| Mean Charlson Score (SD) | 6 (3.2) | 4 (2.8) | **<0.0001^§^** | **1.12 (1.07-1.18)** |
| SCI/D |  |  |  |  |
| No | 393 (80.7) | 361 (64.4) | Reference | --- |
| Yes | 94 (19.3) | 200 (35.6) | **0.43 (0.32-0.57)** | --- |
| *Antibiotic exposure in previous 90 days* |  |  |  |  |
| Any Antibiotics |  |  |  |  |
| No | 342 (70.2) | 350 (62.4) | Reference | Reference |
| Yes | 145 (29.8) | 211 (37.6) | **0.70 (0.54-0.91)** | **0.73 (0.54-0.99)** |
| Fluoroquinolones |  |  |  |  |
| No | 410 (84.2) | 458 (81.5) | Reference | --- |
| Yes | 77 (15.8) | 104 (18.5) | 0.83 (0.60-1.14) | --- |
| 3^rd^ and 4^th^ generation cephalosporins |  |  |  |  |
| No | 476 (97.7) | 547 (97.5) | Reference | --- |
| Yes | 11 (2.3) | 14 (2.5) | 0.90 (0.41-2.01) | --- |
| Carbapenems |  |  |  |  |
| No | 484 (99.4) | 557 (99.3) | Reference | --- |
| Yes | 3 (0.6) | 4 (0.7) | 0.86 (0.19-3.88) | --- |
| *Length of Stay 1 year Prior to Culture* |  |  |  |  |
| Mean, median (SD) | 76 (97.5) | 64 (85.7) | 0.0256^§^ | 1.00 (1.00-1.00) |

Significant associations are shown in bold (p<0.05)

* Other includes, Asian, Native American and Pacific Islander

^†^ other specimen types include wounds, skin/soft tissue, body fluid, bone/joint, rectal cultures

^‡^ respiratory specimen types include bronchial alveolar, tracheal aspirate, induced sputum, etc.

^§^ t-test p-value

| Supplemental Table 2. Bivariate and multivariable analysis of CRPA cultures evaluating association between patient/facility characteristics and 365-day mortality | | | | |
| --- | --- | --- | --- | --- |
| Characteristics  [N=8204] | Death 365 days  After Culture  Date  (%) [N=3303] | No Death 365  Days After  Culture Date  (%) [N=4901] | Unadjusted  Odds  Ratio  (95% CI) | Adjusted Odds  Ratio  (95% CI) |
| *Demographic & Clinical Characteristics* |  |  |  |  |
| Male | 3251 (98.4) | 4751 (96.9) | **1.97 (1.43-2.71)** | --- |
| Age category |  |  |  |  |
| 18-49 | 55 (1.7) | 335 (6.8) | Reference | Reference |
| 50-64 | 622 (18.8) | 1272 (26.0) | **2.98 (2.20-4.02)** | **2.34 (1.70-3.23)** |
| 65+ | 2626 (79.5) | 3294 (67.2) | **4.85 (3.63-6.49)** | **4.10 (3.00-5.59)** |
| Race |  |  |  |  |
| White | 2557 (77.4) | 3865 (78.9) | Reference | Reference |
| African American | 700 (21.2) | 950 (19.4) | 1.11 (1.00-1.24) | --- |
| Other* | 46 (1.4) | 86 (1.7) | 0.81 (0.56-1.16) | --- |
| Ethnicity |  |  |  |  |
| Non-Latine | 3001 (92.5) | 4535 (92.5) | Reference | --- |
| Latine | 302 (9.1) | 366 (7.5) | **1.25 (1.06-1.46)** | --- |
| Region |  |  |  |  |
| South | 1376 (41.7) | 2018 (41.2) | Reference | Reference |
| Midwest | 656 (19.9) | 1048 (21.4) | 0.92 (0.82-1.03) | **0.87 (0.76-0.99)** |
| West | 665 (20.1) | 1103 (22.5) | 0.88 (0.79-1.00) | 0.89 (0.78-1.02) |
| Northeast | 400 (12.1) | 549 (11.2) | 1.07 (0.92-1.24) | 0.89 (0.76-1.05) |
| U.S. Territory | 206 (6.2) | 183 (3.7) | **1.65 (1.34-2.04)** | 1.12 (0.89-1.42) |
| Rurality |  |  |  |  |
| Urban | 3085 (93.4) | 4540 (92.6) | Reference | --- |
| Rural | 218 (6.6) | 361 (7.4) | 0.89 (0.75-1.06) | --- |
| Facility Complexity |  |  |  |  |
| High | 3076 (93.1) | 4410 (90.0) | Reference | --- |
| Low | 227 (6.9) | 491 (10.0) | **0.66 (0.56-0.78)** | --- |
| Care Setting |  |  |  |  |
| Outpatient | 902 (27.3) | 2613 (53.3) | Reference | Reference |
| Long-term care | 420 (12.7) | 623 (12.7) | **1.95 (1.69-2.26)** | **1.23 (1.02-1.48)** |
| Inpatient | 1981 (60.0) | 1665 (34.0) | **3.45 (3.12-3.81)** | **2.17 (1.94-2.43)** |
| Specimen Type |  |  |  |  |
| Urine | 1464 (44.3) | 2859 (58.3) | Reference | Reference |
| Respiratory^‡^ | 1104 (33.4) | 655 (13.4) | **3.29 (2.93-3.69)** | **2.25 (1.98-2.56)** |
| Blood | 114 (3.5) | 70 (1.4) | **3.18 (2.35-4.31)** | **2.05 (1.48-2.85)** |
| Other^†^ | 621 (18.8) | 1317 (26.9) | 0.92 (0.82-1.03) | **0.80 (0.71-0.91)** |
| *Co-Morbidities* |  |  |  |  |
| Mean Charlson Score (SD) | 5 (3.1) | 4 (2.8) | **<.0001^§^** | **1.14 (1.12-1.15)** |
| SCI/D |  |  |  |  |
| No | 2927 (88.6) | 3969 (81.0) | Reference | Reference |
| Yes | 376 (11.4) | 932 (19.0) | **0.55 (0.48-0.62)** | **0.61 (0.52-0.71)** |
| *Antibiotic exposure in previous 90 days* |  |  |  |  |
| Any Antibiotics |  |  |  |  |
| No | 2173 (65.8) | 2681 (54.7) | Reference | Reference |
| Yes | 1130 (34.2) | 2220 (45.3) | **0.63 (0.57-0.69)** | **0.7 (0.63-0.77)** |
| Fluoroquinolones |  |  |  |  |
| No | 2849 (86.2) | 4014 (81.9) | Reference | --- |
| Yes | 454 (13.8) | 887 (18.1) | **0.72 (0.64-0.82)** | --- |
| 3^rd^ and 4^th^ generation cephalosporins |  |  |  |  |
| No | 3203 (97.0) | 4728 (96.5) | Reference | --- |
| Yes | 100 (3.0) | 173 (3.5) | 0.85 (0.66-1.10) | --- |
| Carbapenems |  |  |  |  |
| No | 3283 (99.4) | 4879 (99.5) | Reference | --- |
| Yes | 20 (0.6) | 22 (0.5) | 1.35 (0.74-2.48) | --- |
| *Length of Stay 1 year Prior to Culture* |  |  |  |  |
| Mean, median (SD) | 69 (93.0) | 37 (69.3) | **<0.0001^§^** | **1.00 (1.00-1.00)** |

Significant associations are shown in bold (p<0.05)

* Other includes, Asian, Native American and Pacific Islander

^†^ other specimen types include wounds, skin/soft tissue, body fluid, bone/joint, rectal cultures

^‡^ respiratory specimen types include bronchial alveolar, tracheal aspirate, induced sputum, etc.

^§^ t-test p-value

| Supplemental Table 3. Bivariate/multivariable analysis of CRAB/CRPA blood cultures evaluating association between patient/facility characteristics and 90-day/30-day mortality | | | | | |
| --- | --- | --- | --- | --- | --- |
| Characteristics  [N=402] | Death 90 Days  After Culture  Date  (%) [N=183] | No Death 90  Days After  Culture Date  (%) [N=219] | 90-day Mortality: Unadjusted Odds  Ratio  (95% CI) | 90-day  Mortality:  Adjusted Odds Ratio  (95% CI) | 30-day Mortality: Adjusted Odds Ratio  (95% CI) |
| *Demographic & Clinical Characteristics* |  |  |  |  |  |
| Male | 180 (98.4) | 216 (98.6) | 0.83 (0.17-4.18) | --- | --- |
| Age category |  |  |  |  |  |
| 18-49 | 8 (4.3) | 17 (7.8) | Reference | Reference | Reference |
| 50-64 | 36 (19.7) | 65 (19.7) | 1.18 (0.46-2.99) | 0.86 (0.32-2.32) | 1.46 (0.44-4.87) |
| 65+ | 139 (76.0) | 137 (62.5) | 2.16 (0.90-5.16) | 1.5 (0.60-3.75) | 1.86 (0.60-5.79) |
| Race |  |  |  |  |  |
| White | 137 (74.9) | 145 (66.2) | Reference | --- | --- |
| African American | 46 (25.1) | 71 (32.4) | 0.69 (0.44-1.06) | --- | --- |
| Other* | 0 (0) | 3 (1.4) | <0.001 (<0.001->999.99) | --- | --- |
| Ethnicity |  |  |  |  |  |
| Non-Latine | 153 (83.6) | 195 (89.0) | Reference | --- | --- |
| Latine | 30 (16.3) | 24 (11.0) | 1.59 (0.90-2.84) | --- | --- |
| Region |  |  |  |  |  |
| South | 81 (44.3) | 109 (49.8) | Reference | --- | --- |
| Midwest | 36 (19.7) | 40 (18.3) | 1.21 (0.71-1.07) | --- | --- |
| West | 28 (15.3) | 24 (11.0) | 1.57 (0.85-2.91) | --- | --- |
| Northeast | 16 (8.7) | 27 (12.3) | 0.80 (0.40-1.58) | --- | --- |
| U.S. Territory | 22 (12.0) | 19 (8.7) | 1.56 (0.79-3.07) | --- | --- |
| Rurality |  |  |  |  |  |
| Urban | 173 (94.5) | 204 (93.1) | Reference | --- | --- |
| Rural | 10 (5.5) | 15 (6.9) | 0.89 (0.34-1.80) | --- | --- |
| Facility Complexity |  |  |  |  |  |
| High | 180 (98.4) | 213 (97.3) | Reference | --- | --- |
| Low | 3 (1.6) | 6 (2.7) | 0.59 (0.15-2.40) | --- | --- |
| Care Setting |  |  |  |  |  |
| Outpatient | 32 (17.5) | 52 (23.7) | Reference | Reference | Reference |
| Long-term care | 11 (6.0) | 45 (20.6) | **0.40 (0.18-0.88)** | **0.23 (0.09-0.57)** | 1.86 (0.60-5.79) |
| Inpatient | 140 (76.5) | 122 (55.7) | **1.87 (1.13-3.08)** | 1.63 (0.95-2.78) | **1.78 (1.00-3.16)** |
| Co-Morbidities |  |  |  |  |  |
| Mean Charlson Score (SD) | 6 (3.0) | 5 (3.2) | **0.0017^†^** | **1.11 (1.04-1.19)** | **1.12 (1.04-1.20)** |
| SCI/D |  |  |  |  |  |
| No | 157 (85.8) | 171 (78.1) | Reference | --- | --- |
| Yes | 26 (14.2) | 48 (21.9) | **0.59 (0.35-1.00)** | --- | --- |
| *Antibiotic exposure in previous 90 days* |  |  |  |  |  |
| Any Antibiotics |  |  |  |  |  |
| No | 129 (70.5) | 136 (62.1) | Reference | Reference | Reference |
| Yes | 54 (29.5) | 83 (37.9) | 0.69 (0.45-1.04) | **0.59 (0.37-0.94)** | 0.83 (0.51-1.34) |
| Fluoroquinolones |  |  |  |  |  |
| No | 162 (88.5) | 191 (87.2) | Reference | --- | --- |
| Yes | 21 (11.5) | 28 (12.8) | 0.88 (0.48-1.62) | --- | --- |
| 3^rd^ and 4^th^ generation cephalosporins |  |  |  |  |  |
| No | 180 (98.4) | 210 (95.9) | Reference | --- | --- |
| Yes | 3 (1.6) | 9 (4.1) | 0.39 (0.10-1.46) | --- | --- |
| Carbapenems |  |  |  |  |  |
| No | 182 (99.5) | 216 (98.6) | Reference | --- | --- |
| Yes | 1 (0.5) | 3 (1.4) | 0.40 (0.04-3.84) | --- | --- |
| *Length of Stay 1 year Prior to Culture* |  |  |  |  |  |
| Mean, median (SD) | 91, 47 (107.2) | 79, 36 (100.7) | 0.2681^†^ | 1.00 (1.00-1.00) | 1.00 (1.00-1.00) |
| *Organism* |  |  |  |  |  |
| Acinetobacter | 43 (23.5) | 30 (13.7) | Reference | Reference | Reference |
| Pseudomonas | 140 (76.5) | 189 (86.3) | **0.52 (0.31-0.87)** | **0.46 (0.26-0.81)** | **0.48 (0.27-0.85)** |

Significant associations are shown in bold (p<0.05)

* Other includes, Asian, Native American and Pacific Islander

^†^ t-test p-value

| Supplemental Table 4. Bivariate/multivariable analysis of CRAB/CRPA blood cultures evaluating association between patient/facility characteristics and 365-day mortality | | | | |
| --- | --- | --- | --- | --- |
| Characteristics  [N=402] | Death 365 Days  After Culture  Date  (%) [N=244] | No Death 365  Days After  Culture Date  (%) [N=158] | Unadjusted Odds  Ratio  (95% CI) | Adjusted Odds  Ratio  (95% CI) |
| *Demographic & Clinical Characteristics* |  |  |  |  |
| Male | 155 (98.1) | 241 | 1.56 (0.31-7.80) | --- |
| Age category |  |  |  |  |
| 18-49 | 10 (4.1) | 15 (9.5) | Reference | Reference |
| 50-64 | 55 (22.5) | 46 (29.1) | 1.79 (0.74-4.37) | 1.25 (0.48-3.24) |
| 65+ | 179 (73.4) | 97 (61.4) | **2.77 (1.20-6.40)** | 1.84 (0.76-4.45) |
| Race |  |  |  |  |
| White | 176 (72.1) | 106 (67.1) | Reference | --- |
| African American | 68 (27.9) | 49 (31.0) | 0.84 (0.54-1.30) | --- |
| Other* | 0 (0) | 3 (1.9) | <0.001 (<0.001->999.9) | --- |
| Ethnicity |  |  |  |  |
| Non-Latine | 209 (85.7) | 139 (88.0) | Reference | --- |
| Latine | 35 (14.3) | 19 (12.0) | 1.23 (0.67-2.23) | --- |
| Region |  |  |  |  |
| South | 116 (47.5) | 74 (46.8) | Reference | --- |
| Midwest | 44 (18.0) | 32 (20.2) | 0.88 (0.51-1.51) | --- |
| West | 34 (13.9) | 18 (11.4) | 1.21 (0.63-2.29) | --- |
| Northeast | 24 (9.8) | 19 (12.0) | 0.81 (0.41-1.57) | --- |
| U.S. Territory | 26 (10.7) | 15 (9.5) | 1.11 (0.55-2.23) | --- |
| Rurality |  |  |  |  |
| Urban | 230 (94.3) | 147 (93.0) | Reference | --- |
| Rural | 14 (5.7) | 11 (7.0) | 0.81 (0.36-1.84) | --- |
| Facility Complexity |  |  |  |  |
| High | 239 (97.9) | 154 (97.5) | Reference | --- |
| Low | 5 (2.1) | 4 (2.5) | 0.81 (0.21-3.05) | --- |
| Care Setting |  |  |  |  |
| Outpatient | 23 (9.4) | 33 (20.9) | Reference | Reference |
| Long-term care | 178 (73.0) | 84 (53.2) | 0.67 (0.34-1.32) | **0.35 (0.15-0.81)** |
| Inpatient | 43 (17.6) | 41 (25.9) | **2.02 (1.23-3.33)** | **1.73 (1.00-2.98)** |
| Co-Morbidities |  |  |  |  |
| Mean Charlson Score (SD) | 6 (3.1) | 5 (3.1) | **<0.0001**^†^ | **1.18 (1.09-1.28)** |
| SCI/D |  |  |  |  |
| No | 205 (84.0) | 123 (77.9) | Reference | --- |
| Yes | 39 (16.0) | 35 (22.1) | 0.67 (0.40-1.11) | --- |
| *Antibiotic exposure in previous 90 days* |  |  |  |  |
| Any Antibiotics |  |  |  | --- |
| No | 169 (69.3) | 96 (60.8) | Reference | Reference |
| Yes | 75 (30.7) | 62 (39.2) | 0.69 (0.45-1.05) | **0.59 (0.37-0.94)** |
| Fluoroquinolones |  |  |  |  |
| No | 215 (88.1) | 138 (87.3) | Reference | --- |
| Yes | 29 (11.9) | 20 (12.7) | 0.93 (0.51-1.71) | --- |
| 3^rd^ and 4^th^ generation cephalosporins |  |  |  |  |
| No | 239 (97.9) | 151 (95.6) | Reference | --- |
| Yes | 5 (2.1) | 7 (4.4) | 0.45 (0.14-1.45) | --- |
| Carbapenems |  |  |  |  |
| No | 242 (99.2) | 156 (98.7) | Reference | --- |
| Yes | 2 (0.8) | 2 (1.3) | 0.65 (0.09-4.62) | --- |
| *Length of Stay 1 year Prior to Culture* |  |  |  |  |
| Mean, median (SD) | 91, 46 (109.3) | 74, 34 (94.0) | 0.1134^†^ | **1.00 (1.00-1.00)** |
| *Organism* |  |  |  |  |
| Acinetobacter | 55 (22.5) | 18 (11.4) | Reference | Reference |
| Pseudomonas | 189 (77.5) | 140 (88.6) | **0.44 (0.25-0.79)** | **0.38 (0.20-0.72)** |

Significant associations are shown in bold (p<0.05)

* Other includes, Asian, Native American and Pacific Islander

^†^ t-test p-value
